# Biomimetic Beetle-Inspired Flapping Air Vehicle Actuated by Ionic Polymer-Metal Composite Actuator

**DOI:** 10.1155/2018/3091579

**Published:** 2018-02-27

**Authors:** Yang Zhao, Di Xu, Jiazheng Sheng, Qinglong Meng, Dezhi Wu, Lingyun Wang, Jingjing Xiao, Wenlong Lv, Qinnan Chen, Daoheng Sun

**Affiliations:** ^1^Department of Mechanical and Electrical Engineering, Xiamen University, Xiamen, Fujian, China; ^2^Pen-Tung Sah Institute of Micro-Nano Science and Technology, Xiamen University, Xiamen, Fujian, China

## Abstract

During the last decades, the ionic polymer-metal composite (IPMC) received much attention because of its potential capabilities, such as large displacement and flexible bending actuation. In this paper, a biomimetic flapping air vehicle was proposed by combining the superiority of ionic polymer metal composite with the bionic beetle flapping principle. The blocking force was compared between casted IPMC and IPMC. The flapping state of the wing was investigated and the maximum displacement and flapping angle were measured. The flapping displacement under different voltage and frequency was tested. The flapping displacement of the wing and the support reaction force were measured under different frequency by experiments. The experimental results indicate that the high voltage and low frequency would get large flapping displacement.

## 1. Introduction

Ionic polymer-metal composite (IPMC) is a new type of electroactive polymer material, which can produce large-size deformation under the excitation of electric field [1]. Since the mechanical properties and actuating characteristics of IPMC are very similar to biological muscle, it is also called “artificial muscle” [2]. Notable advantages of IPMC include low driving voltage, relatively large strain, and soft and lightweight mechanisms. It has good prospect and development potential in the fields of bionic robot, sensor, and energy harvesting [3]. In the past, bionic flapping air vehicles were mostly constructed of rigid materials, which were complex, inefficient, and heavy in weight [4, 5]. Due to the unique performance of the IPMC, it is being tried to be applied to flapping mechanism [6]. It is not only easy to control the mechanism by IPMC but also more similar to the biological flexibility [7]. Biomimetic flapping wing mechanisms are used for a deeper understanding of flapping flight [8].

In the last decades, many researchers concentrated on fabrication, modeling, and bionic application of IPMC. He developed an ionic polymer-metal-carbon nanotube composite (IPMCC) actuator composed of a multiwalled carbon nanotube (MWCNT)/Nafion membrane sandwiched between two hybrid electrodes, composed of palladium, platinum, and MWCNTs. The V-I characteristics indicate that the change in shape becomes significant at amplitudes higher than 1.2 V [9]. Chen et al. proposed a novel synthesis technique to fabricate hybrid IPMC membrane actuator capable of generating 3-dimensional (3D) kinematic motions. By controlling each individual IPMC beams, complex 3D motions could be generated [10]. Zhao et al. developed a gradient structure of Nafion in thickness to improve the performance of IPMC. The results of the experiments indicate that the gradient structure would improve the performance both in deformation displacement and blocking force [11]. Caponetto et al. proposed an enhanced fractional-order transfer function (FOTF) model for IPMC membrane working as actuator [12]. He analyzed the effects of the thickness on the performance of IPMC with an electromechanical model. As the thickness increases, the elastic modulus of Nafion membrane and the blocking force of IPMC increase, but the current and the displacement decrease [13]. Shen et al. proposed a hybrid biomimetic underwater vehicle that uses IPMCs as sensors. Propelled by the energy of waves, the underwater vehicle does not need an additional energy source [14]. Shi et al. developed a prototype movable robotic Venus flytrap and evaluated its walking and rotating speeds by using different applied signal voltages [15]. Otis presented the electromechanical characterization of Nafion-Pt microlegs for the development of an insect-like hexapod BioMicroRobot (BMR). BMR microlegs are built using quasi-cylindrical Nafion-Pt ionic polymer-metal composite (IPMC), which has 2.5 degrees of freedom [16]. The thrust performance of a biomimetic robotic swimmer that uses IPMC as a flexible actuator in viscous and inertial flow was studied by Shen et al. A hydrodynamic model based on the elongated body theory was developed [17]. Helical IPMC actuators are newly developed to control the radius of biomedical active stents by Li et al. The helix-shaped IPMC actuator was fabricated through the thermal treatment of an IPMC strip helically coiled on a glass rod. The helical IPMC actuator can be used to realize not only bending motion but also torsional and longitudinal motion [18]. Akle et al. presented the design and development of an underwater jellyfish-like robot using IPMC as propulsion actuators. A water-based IPMC demonstrates a fast strain rate of 1%/s but small peak strain of 0.3% and high current of 200 mA/cm [19]. Lee presented a trade-off design and fabrication of IPMC as an actuator for a flapping device. The internal solvent loss of IPMCs had been conducted for various combinations of cation and solvent in order to find out the best combination of cation and solvent for minimal solvent loss and higher actuation force [20]. Colozza discusses the development of a new aircraft based on a bird's flying principle. Rather than a metal framework covered by riveted plates and hydraulically actuated parts, ionic polymer-metal composite was proposed to be applied to the plane's body and wings [21]. Kim et al. developed a flapping actuator module operated at the resonant frequency by using an IPMC actuator. The performances of the IPMC actuators, including the deformation, blocking force, and natural frequency, were obtained according to the input voltage and IPMC dimensions. The empirical performance model and the equivalent stiffness model of the IPMC actuator are established [22]. Mukherjee and Ganguli used an energy-based variational approach for structural dynamic modeling of the IPMC flapping wing. An optimization study was performed to obtain improved flapping actuation of the IPMC wing. The optimization algorithm leads to a flapping wing with dimensions similar to the dragonfly *Aeshna multicolor*'s wing [23]. With the development of IPMC, it has a wide prospect in bionic robot and other applications. But applying IPMC in flapping air vehicle has lack of study. Due to the unique performance of the IPMC, it can be suitably used in the bionic flapping actuation.

By combing the principle of bionics of beetle flapping, a biomimetic beetle-inspired flapping air vehicle was proposed in this work. The flapping mechanism was fabricated by casted IPMC. The flapping state of beetle-inspired air vehicle was used to analyze the flapping displacement and angle of the wing. The regularity of flapping displacement was investigated under different conditions. Experiments of support reaction force of flapping mechanism were performed and the concept of biomimetic flapping air vehicle actuated by IPMC is shown feasible.

## 2. Beetle-Inspired Flapping Mechanism Design

**Figure 1:**
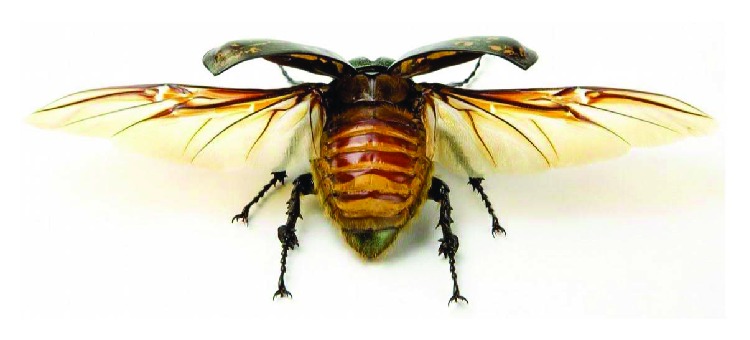
Wings of beetle.

**Figure 2:**
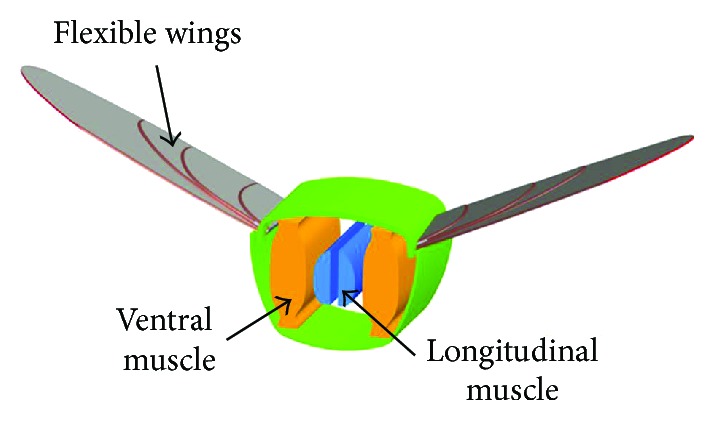
Schematic of beetle flapping bionics.

Beetle flight depends on the control of the chest elastic movement and the force acting on the wings, as shown in Figure 1. The flapping way of the wings is similar to a tuning fork resonance effect. A beetle does not directly flap its wings, but it uses alternating movement of two groups of chest muscle to produce deformation, as shown in Figure 2. Through this way, the wings and chest resonate to produce high-frequency large flapping cycle.

The flapping wings of the insects have two kinds of motions: the longitudinal stroke and the rotation of the wings. In this study, we just consider the stroke of wings [24, 25]. When the wing flaps, the angular velocity of stroking *ω*_s_ is not exactly a simple harmonic motion but a complicated nonlinear motion. In the process of acceleration and deceleration, *ω*_s_(*t*) can be treated as simple harmonic motion.

(1) $$\begin{array}{c} \omega_{s}\left(t\right)\left\{\begin{array}{ll} \omega_{m}\sin\left(\frac{t\pi }{\Delta t_{s}}\right), & t\in \left[0,0.5\Delta t_{s}\right],\\ \omega_{m}, & t\in \left(0.5\Delta t_{s},0.5T-0.5\Delta t_{s}\right],\\ \omega_{m}\sin\left[\left(0.5T-0.5t\right)\frac{\pi }{\Delta t_{s}}\right], & t\in \left(0.5T-0.5\Delta t_{s},0.5T+0.5\Delta t_{s}\right],\\ -\omega_{m}, & t\in \left(0.5T+0.5\Delta t_{s},T-0.5\Delta t_{s}\right],\\ \omega_{m}\sin\left[\left(t-T\right)\frac{\pi }{\Delta t_{s}}\right], & t\in \left(T-0.5\Delta t_{s},T\right], \end{array}\right.\\ \omega_{m}=\frac{\theta_{m}}{\left(2\Delta t_{s}\right)/\left(\pi +0.5T-\Delta t_{s}\right)}, \end{array}$$

where *θ*_m_ is the angle amplitude of flapping wing.

**Figure 3:**
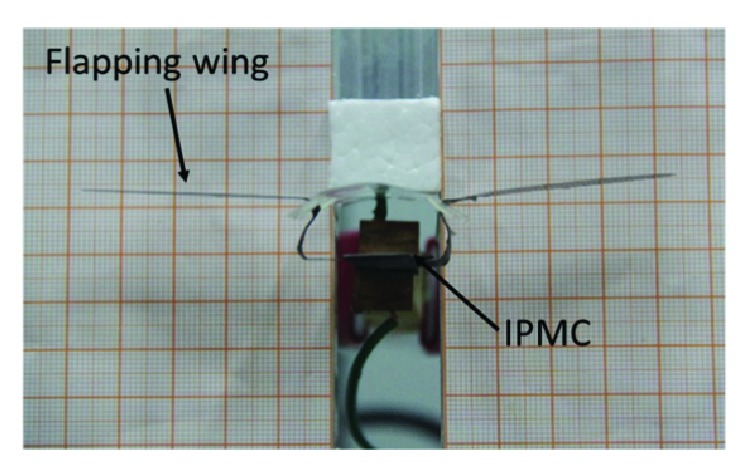
Beetle-inspired flapping mechanism.

In the design process of the beetle-inspired flapping mechanism, a 50 mm long, 10 mm wide, and 420 *μ*m thick IPMC was selected for the actuation because the primary concerns are actuation force and response speed. As shown in Figure 3, the skeleton of flapping mechanism was made of PET film, the wings were made of PVC film, and the size of the wing is 42 mm in length and 15 mm in width. The wing was fixed on the outer surface of PET skeleton by free hinge joint. The IPMC actuator was gripped by a clamp at one side and attached the wings at another side to transfer the actuation force from the IPMC actuator to the wing. Therefore, the bending motion of the IPMC actuator would produce the flapping motion of the beetle-inspired mechanism.

An electromechanical modeling was established for IPMC based on thermodynamics theory [26, 27]. The deformation of IPMC under the combined effect of force field and electric field is as follows:

(2) $$\begin{array}{c} \frac{1}{\rho }=\frac{M}{{YI}_{z}}=\frac{M_{m}+M_{e}}{{YI}_{z}}. \end{array}$$

The moment *M*_m_ by force is described as

(3) $$\begin{array}{c} M_{m}=\frac{{YI}_{z}}{\rho }-M_{e}=\frac{{YI}_{z}}{\rho }-BE, \end{array}$$

where *ρ* is the curvature radius after bending deformation, *M*_m_ is the moment by force, and *M*_e_ is the moment by electrical field. *Y* is the elastic modulus of IPMC and *I*_z_ is the moment of inertia of cross section to *z*-axis. *E* is the electric field and *B* is the bending coefficient of IPMC and is proportional to the square of the length and linearly proportional to the width and thickness of IPMC. Besides, it is also related to the conductivity of the sample and the diffusion rate of the ions used.

## 3. Experiments

### 3.1. Fabrication of Casted IPMC

**Figure 4:**
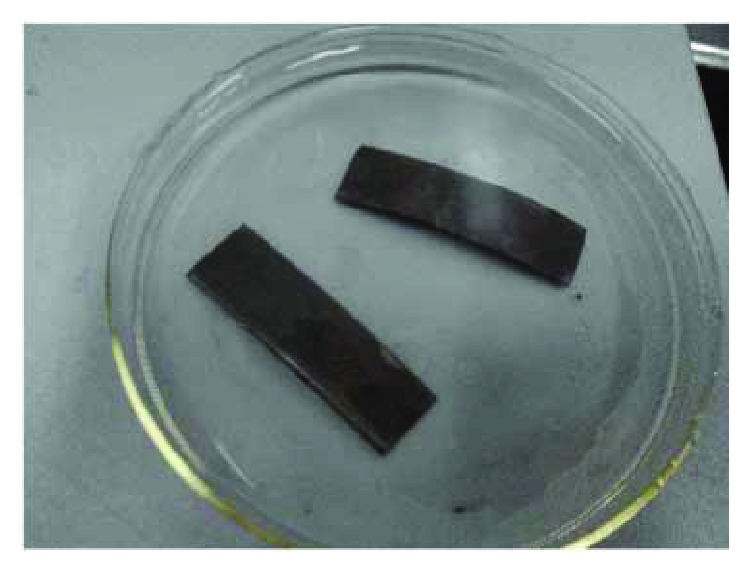
Casted IPMC sample.

The performance of the IPMC varies with its thickness, such as deformation and blocking force. Thick IPMC was chosen for the actuation of the beetle-inspired mechanism. To achieve the desired thick Nafion film, the casting method with Nafion dispersion from DuPont™ was used to fabricate the IPMC in this study. Nafion dispersion and dimethylformamide (DMF) were poured together to cast the Nafion film. The proportion of Nafion and DMF is 4 : 1. The use of DMF is to prevent surface cracks in solidified Nafion during solvent evaporation. The mixed solution was stirred with a magnetic stirrer to make the solution homogeneous. The solution is then placed in a constant-temperature drying oven. The solvent was fully evaporated at 70°C in the oven. It takes almost 18 hours to form the film. The Nafion film was conserved in deionized water. The electrodes of Pt attached to both sides of the Nafion film were fabricated by electroless plating. First, number 1500 sandpaper was used to roughen the surface of the film along one direction. It was used to increase the interfacial area to make the electrode material deposits. Then the film was rinsed chemically with H_2_SO_4_ (0.5%) and H_2_O_2_ (15%) solution, rinsed with boiled deionized water, and dipped into H_2_SO_4_ (0.5%). Second, the film was dipped into the solution of [Pt(NH_3_)_4_]Cl_2_ (3 mg/mm^2^) for about 12 hours to accomplish ion exchange. Third, the platinum complex cations were reduced to the metallic state by using the reducing agents NaBH_4_ (5%); the reaction temperature was from 40 to 60°C. The electrode of Pt was deposited on the surface of the film. Fourth, the film was prepared for the second reduction reaction by rinsing in ultrasonic cleaners after the first reduction reaction. Fifth, the solution of hydrazine hydrate (20%) and the solution of hydroxylammonium chloride (5%) were used to perform the second reduction as the reducing agents. After this reduction, the IPMC sample was fabricated, as shown in Figure 4. Finally, the IPMC sample was rinsed with deionized water and stored in a solution of LiCl for experiment [11].

### 3.2. Experimental Setup

**Figure 5:**
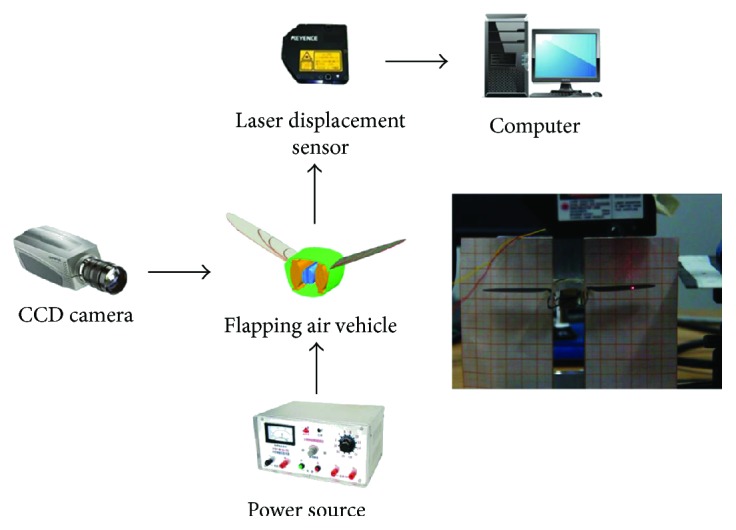
Displacement measurement system.

Since the main performance characteristic of flapping air vehicle is the flapping displacement of the wing, the flapping displacement measurement system was established. The experimental setup of the flapping displacement measurement system is shown in Figure 5. The beetle-inspired flapping air vehicle was placed in front of the coordinate paper (1 mm∗1 mm per grid); the actuated flapping process was captured by digital camera; and the flapping displacement data of the wing was acquired by a laser displacement sensor (LK-080).

**Figure 6:**
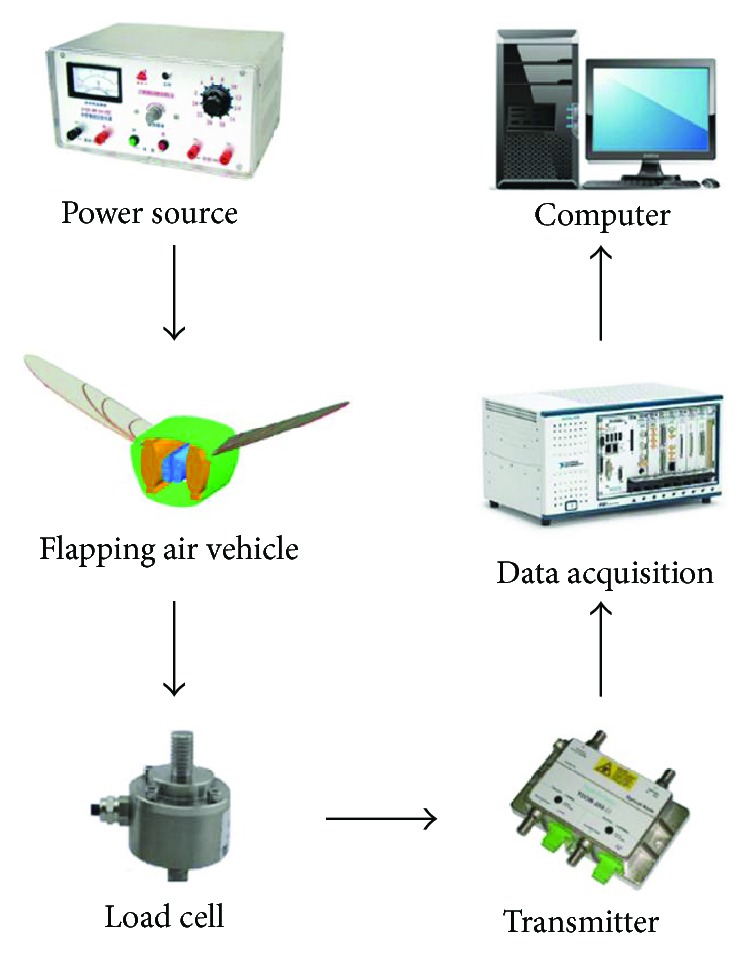
Force measurement system.

The experimental setup of the blocking force measurement system was also established, as shown in Figure 6. The blocking force was measured by a load cell (XH10-5 g) and data acquisition was done by using National Instruments™ PXI system with PXIe-6361 (DAQ).

## 4. Results and Discussion

**Figure 7:**
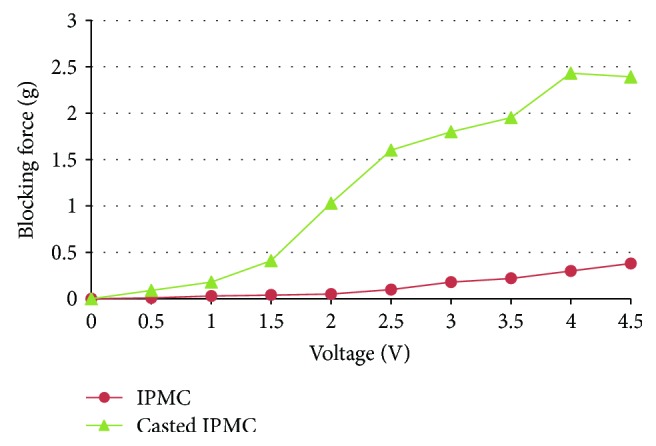
Blocking force of IPMC and casted IPMC.

The IPMC actuator of beetle-inspired air vehicle was fabricated by a casted Nafion membrane. The thickness of IPMC by the casted Nafion was 420 *μ*m. Driven by 0–4.5 V DC, the blocking force of IPMC fabricated by the casted Nafion was compared with IPMC fabricated by a commercial Nafion-117 in Figure 7. It can be found that the blocking force of IPMC by casted Nafion is larger than IPMC fabricated by Nafion-117; the IPMC by casted Nafion can create 2.4 grams of force for 4 V DC. It is suitable for the actuation of a flapping wing than IPMC fabricated by Nafion-117.

**Figure 8:**
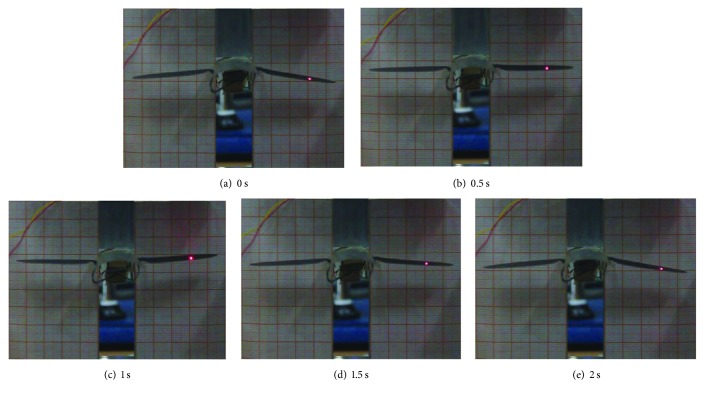
Flapping motion of the beetle-inspired air vehicle.

The wings of the beetle-inspired air vehicle flap in upstroke and downstroke when AC voltage is applied. The front view of the flapping motion of the beetle-inspired air vehicle was recorded by CCD camera. The consecutive snapshots of flapping mechanism is shown in Figure 8. The mechanism was actuated by 4.5 V in a 0.5 Hz frequency sinusoidal wave input voltage. Take one snapshot per 0.5 second. As shown in Figure 8(a), the wings of the mechanism were at the lowest position at 0 second. Then the wings flap in an upstroke position. The highest position of upstroke is at 1 second. After the downstroke of the flapping wings, the wings return to the original position at 2 seconds to finish one upstroke and downstroke cycle. From Figure 8, the maximum tip displacements of the wing is exceeding 10 mm; the maximum flapping angle is 12.5 degrees.

**Figure 9:**
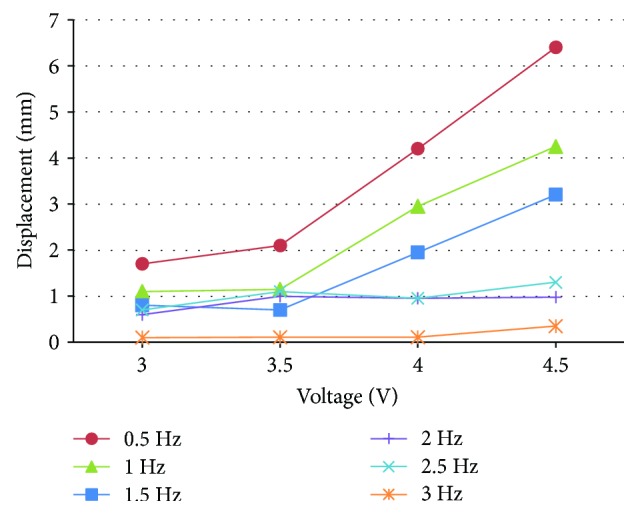
Displacements of the wing under different voltage and frequency.

Figure 9 shows the results of the wing displacements of beetle-inspired air vehicle under different voltage and frequency. The displacements of the wing keep increasing with the increase in the actuation voltage. Meanwhile, the displacements of the wing keep decreasing with the increase in the actuation frequency. The reason is that the driving voltage increases and the blocking force of IPMC increases under the same frequency, so the displacements of the wing generated by the IPMC increase. Under the same driving voltage, the driving frequency decreases and the driving time is lengthened; thus, the displacements increase. It can be seen that the maximum displacement of the wing is obtained under the 4.5 V in 0.5 Hz; the value is 6.4 mm. Similarly, the flapping angle was reduced for higher input frequency.

**Figure 10:**
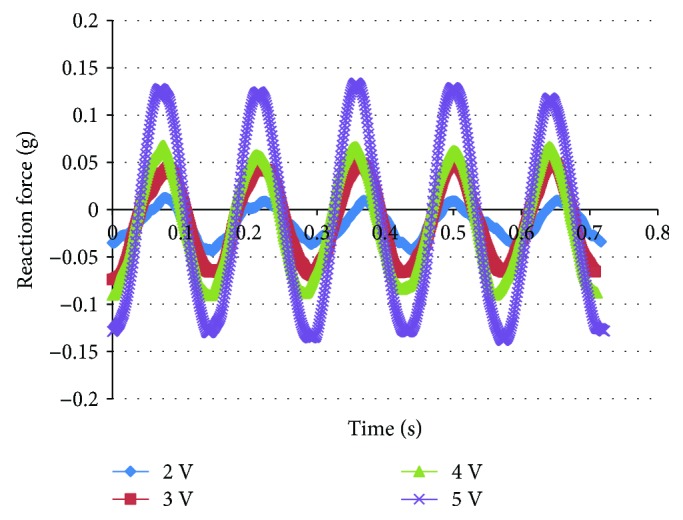
Reaction force of flapping mechanism under 7 Hz, 2–5 V AC.

**Figure 11:**
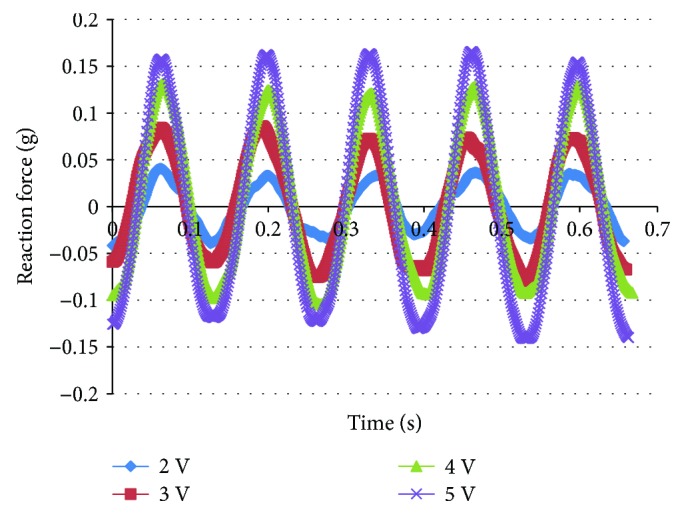
Reaction force of flapping mechanism under 7.5 Hz, 2–5 V AC.

**Figure 12:**
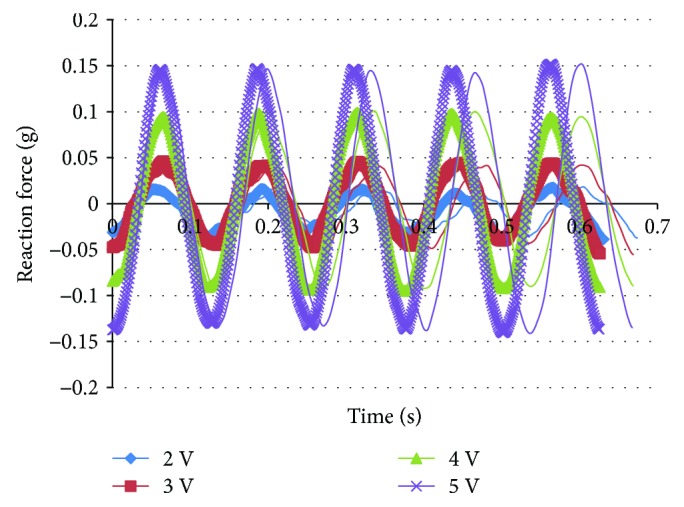
Reaction force of flapping mechanism under 8 Hz, 2–5 V AC.

When the actuation frequency of IPMC is close to the resonant frequency, low-amplitude high-frequency flapping of the wing could be realized. As a result of frequency-sweeping test, the resonant frequency of the IPMC is 7.5 Hz. The measurement of reaction force of the support was carried out at the resonant frequency. Figures 10–12 show the reaction force under a 7 Hz, 7.5 Hz, and 8 Hz sinusoidal input voltage with amplitude varying from 2 to 5 V at 1 V intervals, respectively. With the increase of actuation voltage, the reaction force increases and it also exhibits the regularity of sinusoidal input. As shown in Figure 11, the reaction force under 7.5 Hz is larger than that of 7 Hz and 8 Hz. It indicates that more actuation force and high-frequency flapping could be obtained at the resonant frequency. But it can be seen from the results of the measurement that the actuation force is low when AC voltage is applied, and it is difficult to actuate the flapping wing under high frequency and low voltage.

## 5. Conclusions

In this study, the biomimetic flapping principle of beetle is presented. A beetle-inspired flapping air vehicle is proposed and fabricated by using IPMC actuator of casted Nafion. The thickness of the casted Nafion is 420 *μ*m. The blocking force measurement was carried out to verify the performance of flapping actuation. The flapping state of air vehicle was investigated. The maximum tip displacement and flapping angle were measured. The experiments of displacement test of the flapping wing under different voltage and frequency were investigated. Increasing the voltage and decreasing the frequency would get larger displacements. But it still needs further research for practical use. Future work would be concentrated on the improvement of lift force of the vehicle and the biomimetic pattern of mimicking the flapping wing. The improvement of the performance of IPMC actuator by material modification also needs to be studied.
